# Promising response of proliferative glomerulonephritis with monoclonal IgG deposits to low-dose daratumumab: a case report

**DOI:** 10.3389/fmed.2024.1360979

**Published:** 2024-07-24

**Authors:** Hongyao Xu, Yao Huang, Ling Dong, Hua Yu, Bo Lin

**Affiliations:** ^1^Department of Dermatology, Zhejiang Provincial People’s Hospital Bijie Hospital, Bijie, Guizhou, China; ^2^The Clinical Medical College, Zunyi Medical University, Zunyi, Guizhou, China; ^3^Department of Nephrology, Zhejiang Provincial People’s Hospital Bijie Hospital, Bijie, Guizhou, China; ^4^Department of Cardiovascular Medicine, Zhejiang Provincial People’s Hospital Bijie Hospital, Bijie, Guizhou, China; ^5^Urology & Nephrology Center, Department of Nephrology, Zhejiang Provincial People’s Hospital (Affiliated People’s Hospital, Hangzhou Medical College), Hangzhou, Zhejiang, China

**Keywords:** case report, proliferative glomerulonephritis with monoclonal IgG deposits, daratumumab, MGRS, PGNMID

## Abstract

Proliferative glomerulonephritis with monoclonal immunoglobulin deposits (PGNMID) is a rare disease without standardized treatment modalities. Daratumumab is a human IgG monoclonal anti-CD38 antibody that has been demonstrated to be highly effective and safe in the treatment of PGNMID. This article reports a 66-year-old female who suffered from edema in both lower limbs and face for 6 years with mild proteinuria and hypoproteinemia. Renal biopsy displayed eight glomeruli, among which two presented with glomerulosclerosis, and the remaining six exhibited moderate diffuse hyperplasia of glomerular mesangial cells and stroma with endothelial cell proliferation. Immunofluorescence microscopy revealed lumpy and diffuse deposits of C3, C1q, IgG, and κ light chain in the glomerular mesangium, with strongly positive staining for IgG3 and varying degrees of weak to negative staining for IgG1, IgG2, IgG4, and λ light chain. Additionally, ultrastructural analysis unveiled that the glomerular basement membrane was segmentally thickened, accompanied by diffuse pedicle fusion, segmental tethered insertion, subendothelial deposits, and electron-dense material in tethered areas. The patient received a total dose of 800 mg of daratumumab (400 mg daily for two consecutive days), as well as daily prednisone (25 mg) and valsartan (80 mg), for treatment and achieved complete remission after three-month follow-up. This case represents an early attempt to treat PGNMID with low-dose daratumumab but requires long-term follow-up.

## 1 Introduction

Proliferative glomerulonephritis with monoclonal immunoglobulin deposits (PGNMID) is a disease classified under monoclonal gammopathy of renal significance (MGRS) lesions, first introduced by Nasr in 2004 (1). In this disease, monoclonal immunoglobulins are deposited in glomeruli, causing complement activation and glomerular inflammation and proliferation. However, the specific mechanism underlying monoclonal protein deposition remains to be thoroughly elucidated (2). The clinical features of PGNMID include proteinuria, hematuria, renal insufficiency, and peripheral edema, which are nonspecific.

Unfortunately, the management of PGNMID largely lacks standardized treatment protocols. Conventional therapeutic strategies for PGNMID involve renin-angiotensin system (RAS) blockade and immunosuppression, which have controversial efficacy (2, 3). Researchers have recently observed the astonishing efficacy of clone-directed therapy. Nevertheless, this therapeutic strategy faces a paradoxical challenge in the low detection rate of circulating paraproteins and pathogenic clones in PGNMID patients (4).

Daratumumab, initially approved for the treatment of multiple myeloma, has garnered interest in the field of nephrology because of its mechanism of action targeting CD38-expressing plasma cells. Importantly, a clinical trial at the Mayo Clinic demonstrated that daratumumab also exhibited favorable efficacy in the treatment of PGNMID with an acceptable safety profile, underscoring the necessity to further explore the optimal timing and frequency of daratumumab treatment (5).

In this context, this article reports a case of PGNMID with excellent responses to low-dose daratumumab, providing a reference for future clinical investigations.

## 2 Case presentation

A 66-year-old Chinese female presented to the hospital due to facial and lower limb edema for the past 6 months. The patient weighed 52 kg and was 160 cm in height. Upon admission, her examination showed 0.81 mg/dL serum creatinine, 1.07 g/d proteinuria, 32.9 g/L serum albumin, and the urinary red blood cells of the patient were 2+.

### 2.1 Renal biopsy and hematologic tests

**Figure 1:**
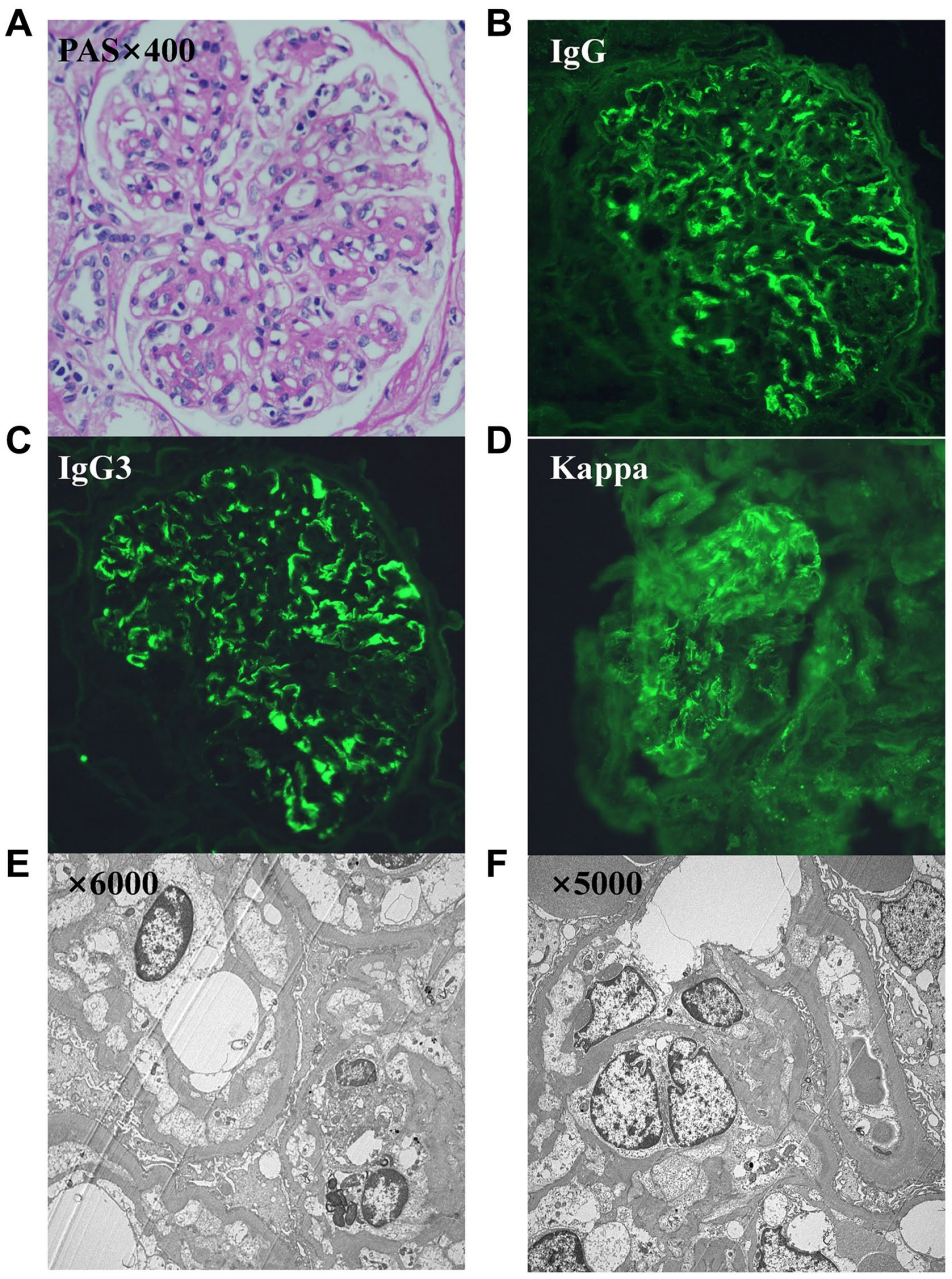
**(A–F)** Images from the renal biopsy. **(A)** Light microscopy exhibits a membranoproliferative GN pattern of injury (periodic acid–Schiff stain, 400x). **(B)** Direct immunofluorescence study shows bright granular IgG. **(C)** Bright granular IgG3. **(D)** Bright κ. **(E,F)** Ultrastructure showed segmental thickening of the glomerular basement membrane, diffuse fusion of pedicles, segmental tethered insertion, and deposition of electron dense material in the subendothelial and tethered areas.

Renal biopsy exhibited eight glomeruli, among which two presented with glomerulosclerosis, and the remaining six showed moderate diffuse hyperplasia of glomerular mesangial cells and stroma with endothelial cell proliferation. Immunofluorescence microscopy indicated lumpy and diffuse deposits of C3, C1q, IgG, and κ light chain in the glomerular mesangium. IgG subclass staining suggested strongly positive staining for IgG3 and weak to negative staining for IgG1, IgG2, IgG4, and λ light chain (Figure 1). Ultrastructural analysis revealed segmental thickening of the glomerular basement membrane, with diffuse pedicle fusion, segmental tethered insertion, subendothelial deposits, and electron-dense materials in the tethered areas (Figure 1). These findings were consistent with IgG3-κ PGNMID. The initial bone marrow biopsy displayed no detectable clones, and flow cytometry of bone marrow illustrated that plasma cells constituted approximately 0.15% of the total nucleated cells, with positive immunophenotyping for CD38, CD138, and CD19. The results of serum protein electrophoresis, serum-free light chain assay, and urinary protein electrophoresis were normal.

### 2.2 B-cell subpopulations and immunophenotypes

**Figure 2:**
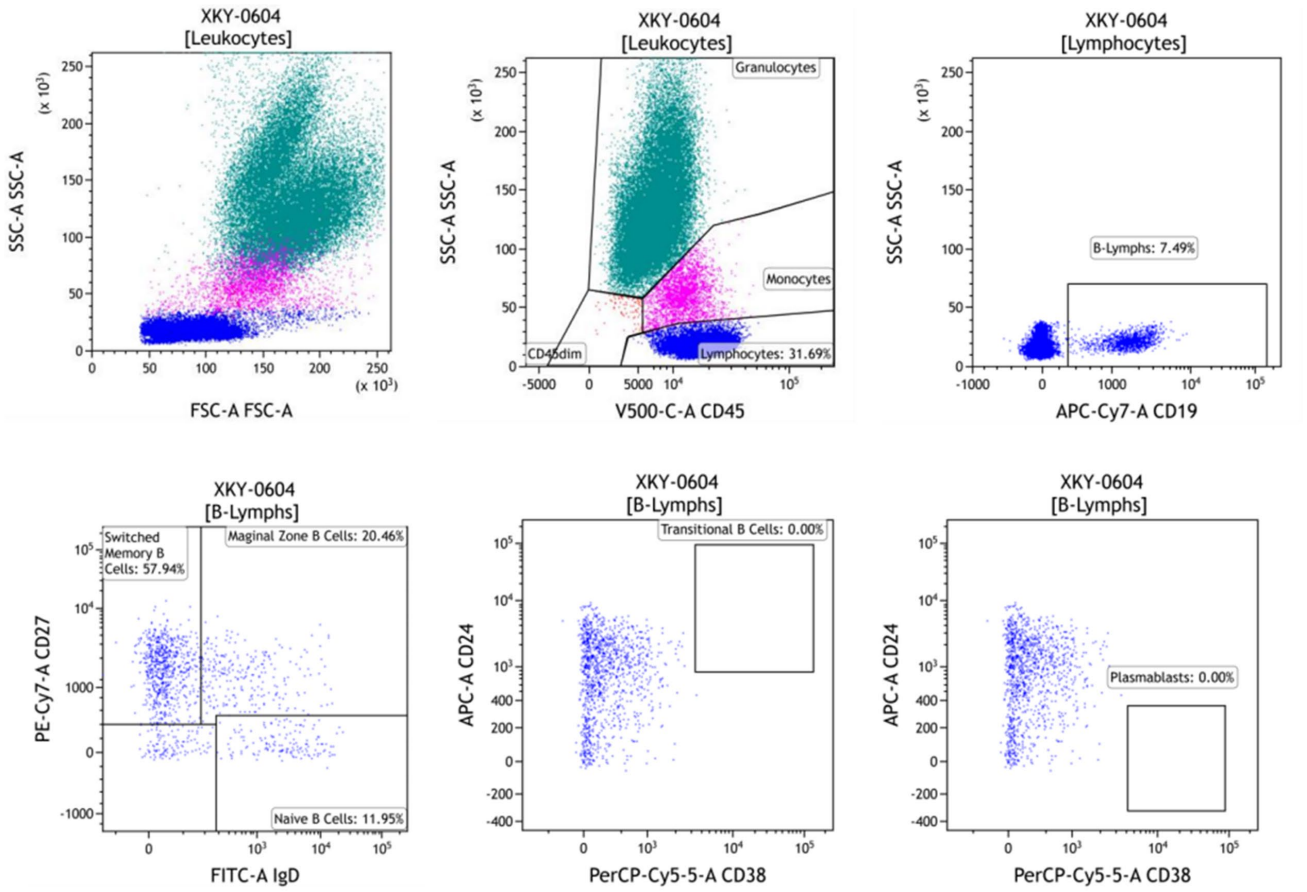
B-cell subset immunophenotyping assessment after 12 weeks of daratumumab treatment. The absolute counts of the CD38 maker were 0 in B cells.

The patient was evaluated for B-cell subsets and immunophenotypes at 2, 6, and 12 weeks of treatment. Results revealed decreases in the absolute number of naive B cells (CD19+, CD27−, and IgD+), marginal zone B cells (CD19+, CD27−, and IgD+), memory B cells (CD19+, CD27+, and CD38−), transitional B cells (CD19+, CD24+, and CD38+), and plasmablasts (CD19+ and CD38+). Notably, the absolute counts of transitional B cells (CD19+, CD24+, and CD38+) and plasmablasts (CD19+ and CD38+) were undetectable in both the second and third evaluations (Figure 2).

### 2.3 Treatment

The patient had a history of hypertension for over 10 years, which was managed by oral administration of valsartan (80 mg daily). After the diagnosis of PGNMID, the patient received valsartan (80 mg daily) and prednisone (50 mg daily) according to recommendations from the International Kidney and Monoclonal Gammopathy Research Group due to the initial presentation of 0.81 mg/dL serum creatinine and 1.07 g/d urine protein, which is suggestive of stage 2 chronic kidney disease (CKD). However, after a month, proteinuria and serum creatinine levels were not improved. Given that the recommended dose of daratumumab is 16 mg/kg, she received a total dose of 800 mg of daratumumab (400 mg daily for two consecutive days). Subsequent to daratumumab treatment, the dose of prednisone was halved (25 mg daily) and the dose of valsartan remained unchanged. Prior to each infusion, the patient was intravenously injected with 20 mg of methylprednisolone sodium succinate and 1 g of calcium gluconate and also intramuscularly injected with 25 mg of promethazine hydrochloride. Daratumumab treatment was completed successfully without any adverse reactions, including allergies. After 2 weeks of treatment, however, she developed a pulmonary infection and was discharged following aggressive anti-infection treatment.

### 2.4 Follow-up

**Figure 3:**
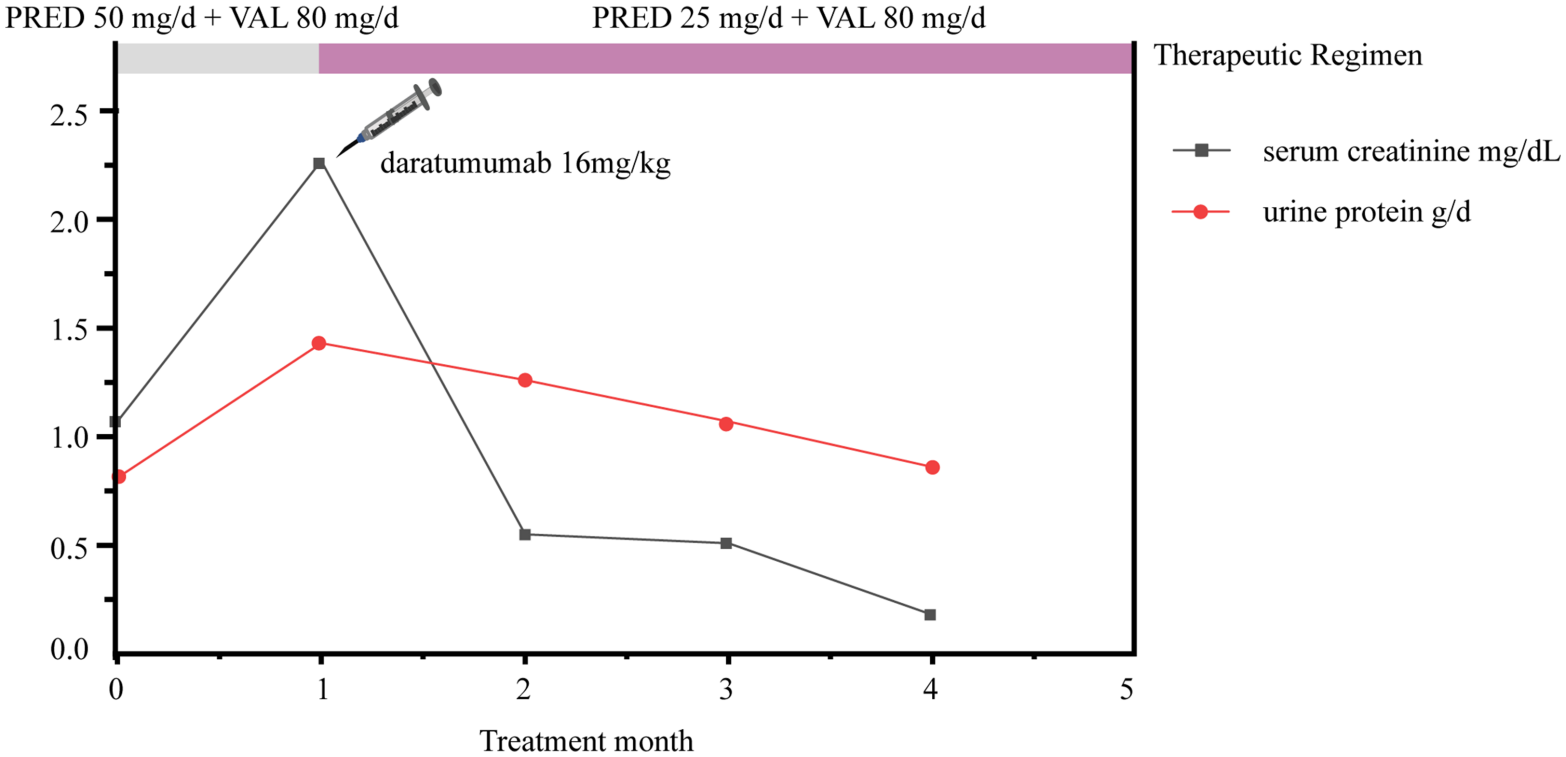
Trends in patient urine protein and serum creatinine changes. PRED, prednisone; VAL, valsartan.

After 1 month of treatment with steroids combined with valsartan (prior to daratumumab treatment), urinary red blood cells and proteinuria increased to 3+ and 2.27 g/d, respectively. Fortunately, urinary red blood cells decreased to 2+ at 2 weeks after daratumumab treatment and were weakly positive at 16 weeks. Moreover, the edema of the patients completely subsided after 1 month of treatment with daratumumab plus half-dose steroids and 80 mg of valsartan, with 0.55 g/d proteinuria and 1.26 mg/dL serum creatinine (Figure 3). After 3 months of treatment, further improvements were observed, as proteinuria was reduced to 0.18 g/d, serum creatinine decreased to 0.86 mg/dL (Figure 3), and the absolute count of CD38 marker in B cells was reduced to zero. Unfortunately, the patient’s treatment status and prognosis remained unknown due to the loss of follow-up. However, on May 2024, the patient re-visited our hospital presenting with facial and lower limb edema. Upon admission, laboratory examinations revealed a serum creatinine level of 1.28 mg/dL, proteinuria level of 1.24 g/d, serum albumin level of 33.2 g/L, and urinalysis showed the presence of red blood cells (3+). Based on the patient’s medical history and these recent laboratory examination results, we suspected that the PGNMID disease of this patient has progressed. Thus, the subsequent treatment plan is currently being formulated. The patient is still being followed up.

## 3 Discussion and conclusions

PGNMID is a unique form of MGRS in which IgG deposition in the glomeruli leads to renal injury. IgG3 is the most frequently deposited IgG subclass. Different from other subclasses, IgG3 possesses higher charge and molecular mass, can self-aggregate through Fc-Fc interactions, and has a strong complement-fixing capacity (6).

In PGNMID patients receiving either RAS inhibitors or immunosuppressive therapy, the renal remission rate is suboptimal, with a poor prognosis. The retrospective study by Nasr in 2009 unveiled that among 9 patients treated with RAS blockade, only 2 achieved complete remission (CR) (22%), 2 patients achieved partial remission (PR) (22%), and 1 patient progressed to end-stage renal disease (ESRD). Additionally, there were only 2 patients with CR (11%), 6 patients with PR (33%), and 3 patients progressing to ESRD among 18 patients receiving immunomodulatory (IM) drugs such as prednisone, alkylating agents, bortezomib, thalidomide, lenalidomide, cyclosporine, and rituximab (2). The primary goal of PGNMID management is to protect and reverse renal function and prevent repeat kidney transplantation. In case of compromised renal function or biopsy features suggestive of condition deterioration, PGNMID should be treated by IM therapy with the use of common agents such as steroids, cyclophosphamide, mycophenolate mofetil, rituximab, thalidomide, and bortezomib (3). Recently, Gumber et al. observed superior efficacy of clone-directed therapy over traditional treatment in the treatment of PGNMID (4). However, clone-directed therapy is confronted with the challenge that circulating paraproteins and pathogenic clones cannot be detected to guide treatment selection for PGNMID. A prior study unraveled that only about 35% of circulating paraproteins and only approximately 40% of pathogenic clones were detected in bone marrow biopsy. In such circumstances, empirical chemotherapy is predominantly used for the treatment of PGNMID at present (7). Given that IgG is the most commonly deposited subclass in PGNMID, anti-plasma cell agents are recommended.

Gumber et al. and the International Kidney and Monoclonal Gammopathy Research Group advocate chemotherapy for PGNMID patients with stage 3 or higher CKD. Tailoring chemotherapy regimens to the underlying clone is crucial to improving treatment outcomes (4, 8). The study by Guiard et al. further demonstrated that rituximab-based regimens were superior in PGNMID patients with CD20-positive potential B-cell clones, as evidenced by 5 with CR and 2 with PR among 7 patients treated with rituximab, with better efficacy than traditional treatment or the Cyclophosphamide/Bortezomib/Dexamethasone regimen (9). Daratumumab is a human IgG monoclonal anti-CD38 antibody, which exerts its effect of targeting plasma cells through direct action or Fc-dependent and immunomodulatory mechanisms of plasma cell depletion (10). In a phase 2 trial, ten PGNMID patients received at least one dose of daratumumab (16 mg/kg), who all showed renal responses during the 12-month follow-up, with 4 patients with CR and 6 patients with PR. In 162 infusions, five serious adverse events occurred, of which two were severe infectious complications (5). Intriguingly, the proteinuria and serum creatinine of a patient were significantly improved after only one infusion of daratumumab, which was sustained until 12 months. Hence, a short duration of therapy may be a reasonable strategy for managing PGNMID. Correspondingly, the research by Almaani unveiled the positive clinical efficacy of daratumumab, with three out of five patients displaying renal responses (11). Overall, daratumumab is a viable alternative for the treatment of PGNMID. However, further studies are warranted to determine the optimal dosing frequency of daratumumab.

For our patient, we regularly monitored B-cell subpopulations and immunophenotyping and guided the use of daratumumab based on the absolute count of CD38 markers in B cells. The patient achieved complete remission, with the absolute count of CD38 markers reduced to zero. As a result, the administration of daratumumab was discontinued. In conclusion, this case underlines the potential of low-dose daratumumab plus low-dose prednisone as a viable option for refractory PGNMID patients. Due to limited domestic and international reports, additional systematic clinical trials are required to optimize the use of daratumumab in PGNMID treatment.

## Data availability statement

The original contributions presented in the study are included in the article/supplementary material, further inquiries can be directed to the corresponding author.

## Ethics statement

The studies involving humans were approved by Zhejiang Provincial People’s Hospital Bijie Hospital Ethics Committee (Affiliated Zhejiang Provincial People’s Hospital Bijie Hospital). The studies were conducted in accordance with the local legislation and institutional requirements. The participants provided their written informed consent to participate in this study. Written informed consent was obtained from the individual(s) for the publication of any potentially identifiable images or data included in this article.

## Author contributions

HX: Investigation, Writing – original draft, Writing – review & editing. YH: Investigation, Project administration, Writing – original draft. LD: Writing – original draft, Writing – review & editing. HY: Investigation. BL: Conceptualization, Investigation, Project administration, Writing – review & editing, Writing – original draft.
